# Counting hydrogen in porous adsorbents

**DOI:** 10.1080/14686996.2026.2722037

**Published:** 2026-08-25

**Authors:** Susmita Roy, Takahiro Kondo

**Affiliations:** a Department of Materials Science, Institute of Pure and Applied Sciences, University of Tsukuba, Tsukuba, Ibaraki, Japan; b Advanced Institute for Materials Research (WPI-AIMR), Tohoku University, Sendai, Miyagi, Japan; c Hydrogen Boride Research Center, Tsukuba Institute of Advanced Research, University of Tsukuba, Tsukuba, Ibaraki, Japan; d Research Center for Energy and Environmental Materials, National Institute for Materials Science, Tsukuba, Ibaraki, Japan

**Keywords:** Hydrogen storage, physisorption, porous adsorbents, Gibbs excess, total uptake, volumetric capacity, counting hydrogen

## Abstract

The ultimate goal of the hydrogen economy is to develop a hydrogen storage system that efficiently meets or exceeds the targeted goal set by the US Department of Energy (DOE) for onboard light-duty vehicles. These targets are defined in terms of usable quantity of hydrogen deliverable to the fuel cell system, including all stored hydrogen, media, reactants, and system components. To achieve reliable system performance, it is essential to precisely and uniformly measure the material properties. However, there have been several discrepancies and methodological inconsistencies, particularly in the reporting of volumetric storage capacity of porous frameworks. In the case of porous systems, where hydrogen is stored through the physisorption process on the pore surface, the concepts of ‘*excess*’, ‘*absolute*’, and ‘*total*’ hydrogen storage appear. Therefore, estimation of storage performance in such systems is complex. Many porous materials have been investigated over the years; however, without the assurance of uniform and proper methodological considerations, the reported performance of these materials remains difficult to assess reliably and does not provide the benefit toward the work in hand. In this perspective, we address these issues and propose certain reporting protocols to ensure valid identification of suitable materials for further development.

## IMPACT STATEMENT

This perspective clarifies fundamental concepts of ‘counting’ hydrogen in porous adsorbents by reconciling *excess*, *absolute*, and *total* uptake with clear definitions of volume and density for reliable comparison and meaningful assessment for practical hydrogen-storage materials.

## GRAPHICAL ABSTRACT

**Figure:**
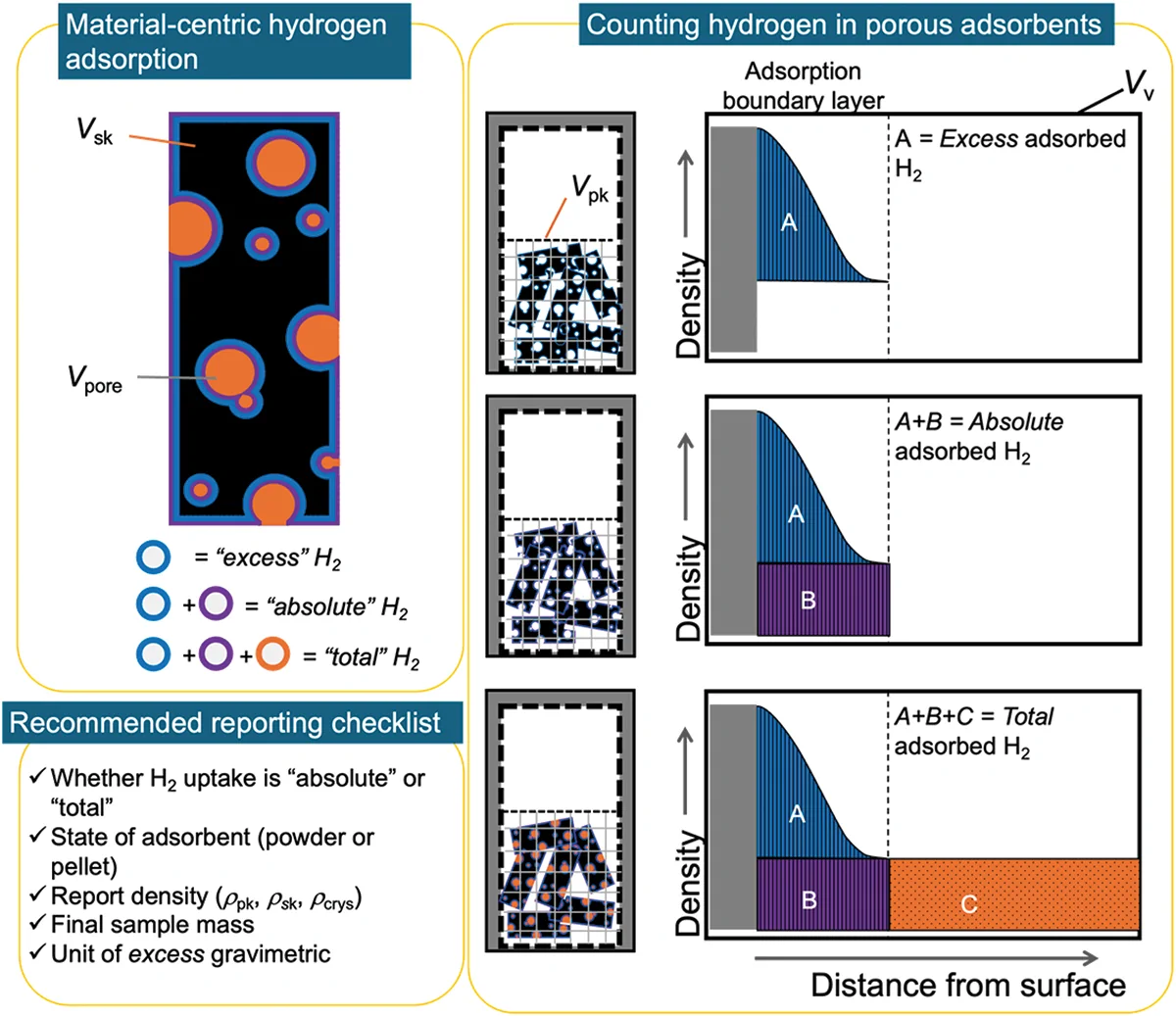

## 1. Why do definitions matter?

The ultimate DOE target for onboard hydrogen storage in light-duty vehicles specifies a usable gravimetric capacity of 0.065 kg H_2_kg^–1^ system, a usable volumetric capacity of 0.050 kg H_2_L^−1^system, with a delivery pressure range of 5 to 12 bar and an operating temperature range of −40 to 80°C [1]. Against this benchmark, when a researcher reports that a porous metal-organic framework ‘stores 7.5 wt% or 48 g L^−1^ of hydrogen at 77 K and 100 bar’, several critical queries arise: How is hydrogen being counted within the porous adsorbent? Is the reported uptake based on the adsorbent mass alone, or does it account for the total system weight? What is the physical form of the adsorbent – loose powder, compacted pellet, or crystal unit cell? And precisely under which conditions is the uptake reported – a deliverable pressure swing (*P*-swing) or a pressure-temperature swing (*PT*-swing)?

Porous adsorbents such as metal-organic frameworks (MOFs), covalent-organic frameworks (COFs), porous carbons, and zeolites are low-density and highly porous adsorbents with high surface area, which have attracted considerable attention as promising candidates for hydrogen storage over the past few decades [2–6]. Hydrogen is typically physisorbed on the surface of the pore wall of such adsorbents, and the quantity is directly obtained as ‘*Gibbs excess*’ amount from a calibrated gravimetric or manometric experiment. At elevated pressure, a substantial fraction of hydrogen associated with the adsorbent is present as a compressed fluid in gas-accessible pores and void spaces. Consequently, quantifications of hydrogen require an accurate definition and measurement of the gas-accessible volume assigned to the material. However, across several papers, the choice of counting hydrogen is presented differently, which creates difficulty in comparing the reported results. Additionally, methodological inconsistencies create a significant barrier to understanding for those new to the field.

Density is defined as mass per unit volume. For porous materials, however, the numerical value of density depends on which volume is used. Therefore, density introduces a second source of ambiguity. Highly porous crystals can have excellent hydrogen uptake per unit mass but possess low packing density. Densification can improve volumetric performance; it alters the density of the material from the ideal crystal density. So, several key volume and density terms are required to describe powders, pellets, monoliths and other packed forms in the container, as they form the foundation upon which hydrogen uptake calculations are set.

It is therefore essential to establish a clear understanding of how hydrogen uptake is defined, measured, and counted in adsorbents. This Perspective reconciles the principal conventions discussed by Parilla *et al*. [7], Samantaray *et al.* [8], and the DOE Best Practices report [9], together with experimental approaches such as that of Villajos [4] and Furukawa *et al*. [10]. It should be noted that the definitions used to count hydrogen and evaluate the performance strongly depend on the selected figures of merit (FOMs). FOMs are defined for specific objectives and may refer either to the hydrogen-storage material itself or to the complete storage system.

## 2. Definitions of adsorbent volume and density

For porous materials, a useful starting point is to understand the volume definitions (Figure 1). The inclusion of certain volume terms depends on the selected FOM (whether performed at the materials and system levels). For each volume definition considered here, a corresponding density can also be defined such that,

(1) $$\rho_{s,t}=\;\frac{m_{s}}{V_{t}}$$

**Figure 1.**
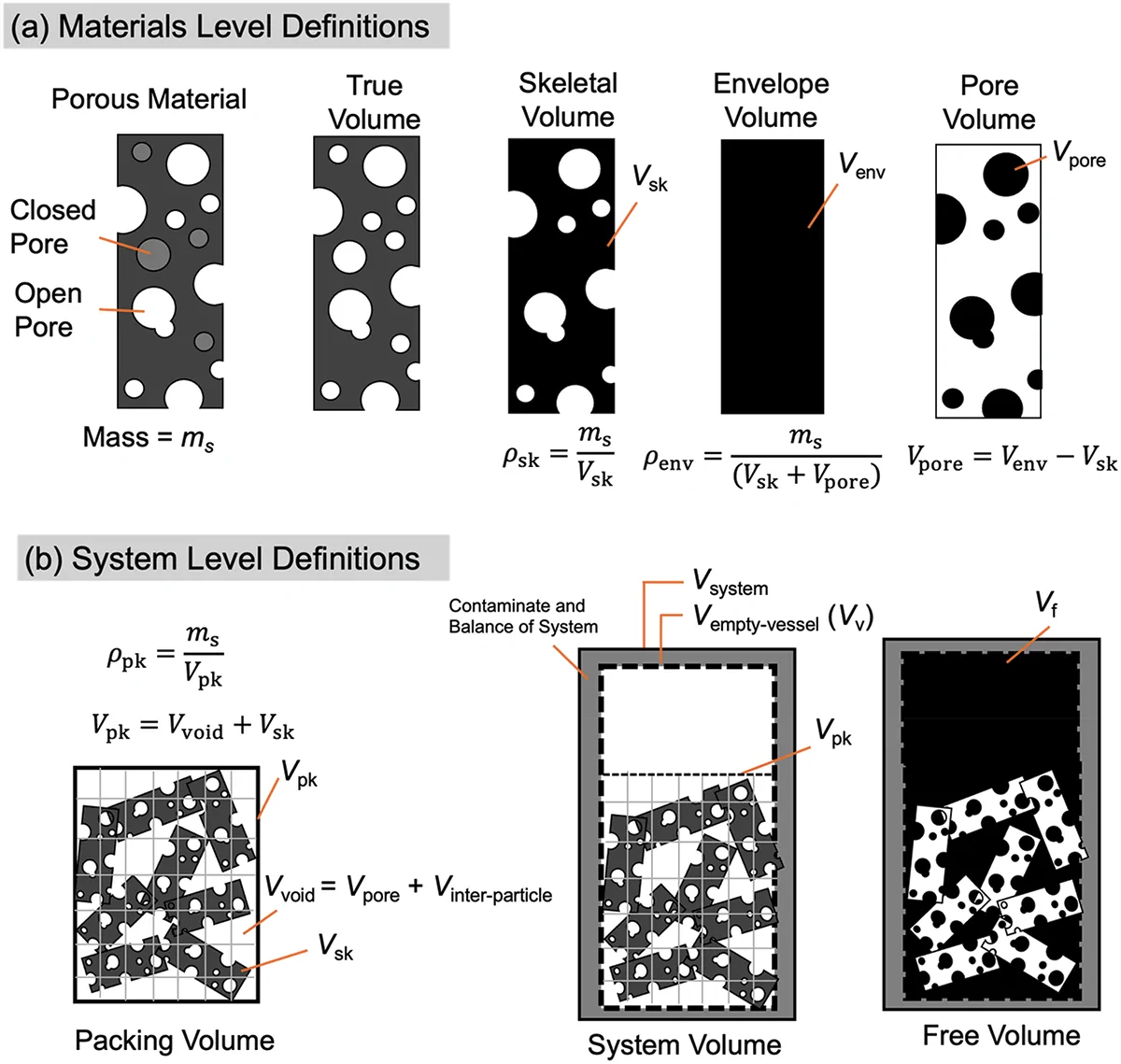
Volume and density definitions: illustration of different kinds of volume concepts and considerations relevant to the hydrogen storage capacities calculations at (a) a materials level and (b) system level. Reproduced from Figure 84 of the ‘best practices’ document, K. Gross, Ref [9].

Where the mass(es) ‘s’ and associated volume(s) ‘t’ must be specified explicitly.

The definitions below assume a porous material containing gas-accessible open pores, while the solid framework and closed pores are inaccessible to the probe gas. Selected volume definitions are illustrated schematically in Figure 1(a) to clarify the distinctions among the different volume and density terms.

### 2.1. Skeletal volume and skeletal density

The skeletal volume, *V*_sk,_ referred to as gas-inaccessible or absolute volume, is the volume occupied by the solid framework together with closed pores. The corresponding skeletal density, *ρ*_sk_, is the mass of the adsorbent divided by this gas-inaccessible volume and therefore excludes open, gas-accessible pores.

(2) $$\rho_{\mathrm{sk}}=\;\frac{m_{s}}{V_{\mathrm{sk}}}$$

*V*_sk_ is commonly determined by using helium pycnometry or a volumetric pressure-composition-temperature (PCT) instrument, typically with gas-displacement volume of the sample using helium as probe at elevated pressure and room temperature. This determination assumes that helium accesses all H_2_-accessible open pores and that helium adsorption, non-isothermal effects, gas impurities, and real-gas behavior are adequately treated. Because *V*_sk_ enters both manometric headspace calibration and gravimetric buoyancy correction, uncertainty in *V*_sk_ directly affects the measured excess isotherm. The measurement method, pressure range, and temperature should therefore be reported.

### 2.2. Idealized crystal volume and crystal density

For highly crystalline porous adsorbents, the crystallographic unit cell defines an ideal single-crystal volume, *V*_crys_, that contains both the framework skeleton and intrinsic intracrystalline pores (*V*_pore_). The respective crystal density, *ρ*_crys_ = *m*_s_/*V*_crys_, represents an upper-density limit associated with perfect crystal packing and provides a reference for comparison with the actual density of powders, pellets, or monoliths [11,12].

### 2.3. Envelope volume and envelope density

The envelope volume (*V*_env_) or apparent volume is defined as the volume of particles enclosed by an external boundary of a particle, pellet, or monolith. It only includes solid skeleton and intraparticle pores but excludes the interparticle spaces.

(3) $$\mathrm{Thus},\;\;\;V_{\mathrm{pore}}=V_{\mathrm{env}}-V_{\mathrm{sk}}=m_{s}\left(\frac{1}{\rho_{\mathrm{env}}}-\frac{1}{\rho_{\mathrm{sk}}}\right)$$

(4) $$\;\;\;\;\;\;\;\;\;\;\;\;\;\;\;\;\;\;\;\;\;\;\;\;\;\;\;\;\;\;\;\;\;\;\;\;\;\;\;\;\;\;\;\mathrm{or},\;\;\;\;\;\;\;\;\;V_{\mathrm{env}}=V_{\mathrm{pore}}+V_{\mathrm{sk}}$$

For a porous crystalline framework with permanent intrinsic pores, *V*_crys_ is conceptually similar to *V*_env_ because the pore volume is included within the overall crystal.

### 2.4. Packing volume and packing density

This packing volume, *V*_pk,_ is the volume occupied by a known quantity of material (powder, pellets or monoliths), when loaded into a container under a specified packing procedure such as tapping, pressing or jolting, etc. It is the sum of H_2_ accessible void volume (*V*_void_) associated with packing bed and *V*_sk_ (inaccessible to H_2_), as expressed below:

(5) $$V_{\mathrm{pk}}=\;V_{\mathrm{pore}}+\;V_{\mathrm{inter}-\mathrm{particle}}+\;V_{\mathrm{sk}}=\;V_{\mathrm{void}}+\;V_{\mathrm{sk}}$$

(6) $$V_{\mathrm{void}}=\;V_{\mathrm{pore}}+\;V_{\mathrm{inter}-\mathrm{particle}}=\;V_{\mathrm{pk}}-\;V_{\mathrm{sk}}$$

The respective density is called packing density, *ρ*_pk_ = *m*_s_/ *V*_pk_. It should be noted that *ρ*_pk_ depends on several factors such as particle size, size distribution, electrostatic interaction between particles, external pressure to aggregate pellets, tapping method, binder content etc (as can be seen in Table 1) [8,13,15].

**Table 1.** Powder and densified powder properties of porous adsorbents. Here, a benchmark MOF *viz* MOF-5 is chosen to show the variation in the density when packed differently in a container. ENG additives mentioned in the sample are expanded natural graphite, used to improve the thermal conductivity in MOF-5.

Sample	Form	*ρ*_pk_ (g cm^−3^)	*ρ*_sk_ (g cm^−3^)	*ρ*_crys_ (g cm^−3^)	*V*_pore_ (cm^3^ g^−1^)	BET SA (m^2^ g^−1^)	$\chi_{\mathrm{void}}$ (%)	Reference
MOF-5	Powder Tapped	0.13	2.03	–	1.27	2763	94	[8,13]
MOF-5	Powder Jolted	0.22	2.03	–	1.27	2763	89	[8,13]
MOF-5	Pellet	0.52	2.03	–	1.12	2263	74	[8,14]
MOF-5/ENG 1%	Pellet	0.49	2.03		1.12	2584	76	[8,14]
MOF-5/ENG 5%	Pellet	0.47	2.03		1.14	2623	77	[8,14]
MOF-5/ENG 10%	Pellet	0.48	2.03		1.06	2413	76	[8,14]
MOF-5 crystal N_2_ probe	Powder	–	2.549	0.5992	1.2765	3633	76.59	[8]
MOF-5 crystal H_2_ probe	Powder	–	2.7334	0.5992	1.3030	3733	78.08	[8]

For volumetric hydrogen uptake, it is important to know the gas-accessible volume of the selected control volume, *V*_void_, and the corresponding gas-accessible void fraction $\left(\chi_{\mathrm{void}}\right)$ within the packed volume, as expressed by Equations (7) and (8):

(7) $$V_{\mathrm{void}}=\;m_{s}\left(\frac{1}{\rho_{\mathrm{pk}}}-\frac{1}{\rho_{\mathrm{sk}}}\right)$$

and,

(8) $$\chi_{\mathrm{void}}=\frac{V_{\mathrm{void}}}{V_{\mathrm{pk}}}=1-\frac{\rho_{\mathrm{pk}}}{\rho_{\mathrm{sk}}}$$

These Equations (6)**–**(8) are the central to calculate the total hydrogen uptake especially in a packed bed.

The term ‘packing density’ sometimes also referred to as ‘bulk density’ for powder sample packing, whereas ‘envelope density’ is used when a pellet sample is packed in the container. Therefore, readers must carefully consider these terms and respective uptake calculation while reporting [8,9,14].

### 2.5. Instrument free volume

In a manometric instrument, the free or dead volume (*V*_f_) is the calibrated internal volume accessible to the gas after the sample is loaded. Depending on the calibration condition, this volume includes the sample pores, intraparticle voids, and any unused headspace in the sample cell and connecting regions included during calibration (as shown in Figure 1(b)).

(9) $$V_{f}=\;V_{v}-\;V_{\mathrm{sk}}$$

## 3. Counting hydrogen in porous adsorbents

In porous adsorbents, H_2_ doesn’t penetrate the interior of the adsorbent; rather, it is adsorbed near the surface through physisorption interaction. The important elements pertaining to physisorbed H_2_ storage by a solid adsorbent (s), the adsorbate (a) and the adsorptive gas (g) are illustrated for a simple Gibbs *excess* schematic diagram as shown in Figure 2. The adsorptive gas phase is described by a real gas equation of state $\rho_{g}\left(H_{2},P,T\right)$, as a function of pressure (P) and temperature (T), where $\rho_{g}\left(H_{2}\right)$ is the H_2_ density in a storage container in the absence of any sorbent interactions. The volume of sorbent materials, where gas cannot penetrate, is referred to as *V*_sk_. The *V*_f_ or ‘dead volume’ is the difference between the instrument vessel volume, *V*_v_ (for the storage system level, the total storage tank *V*_empty-vessel_ volume, as shown in Figure 1(b)) and the material’s *V*_sk_. The adsorbed phase volume (*V*_ads_) denotes a conceptual near-surface region in which H_2_ molecules are influenced predominantly by the adsorbent interaction potential. The hydrogen density is enhanced near an adsorbing surface and gradually approaches the bulk-fluid density with increasing distance from the surface. Because there is no abrupt physical boundary between an adsorbed layer and free gas, hydrogen counting depends on the chosen reference state and control volume. Figure 2 separates the density profile into regions A, B, and C.

**Figure 2.**
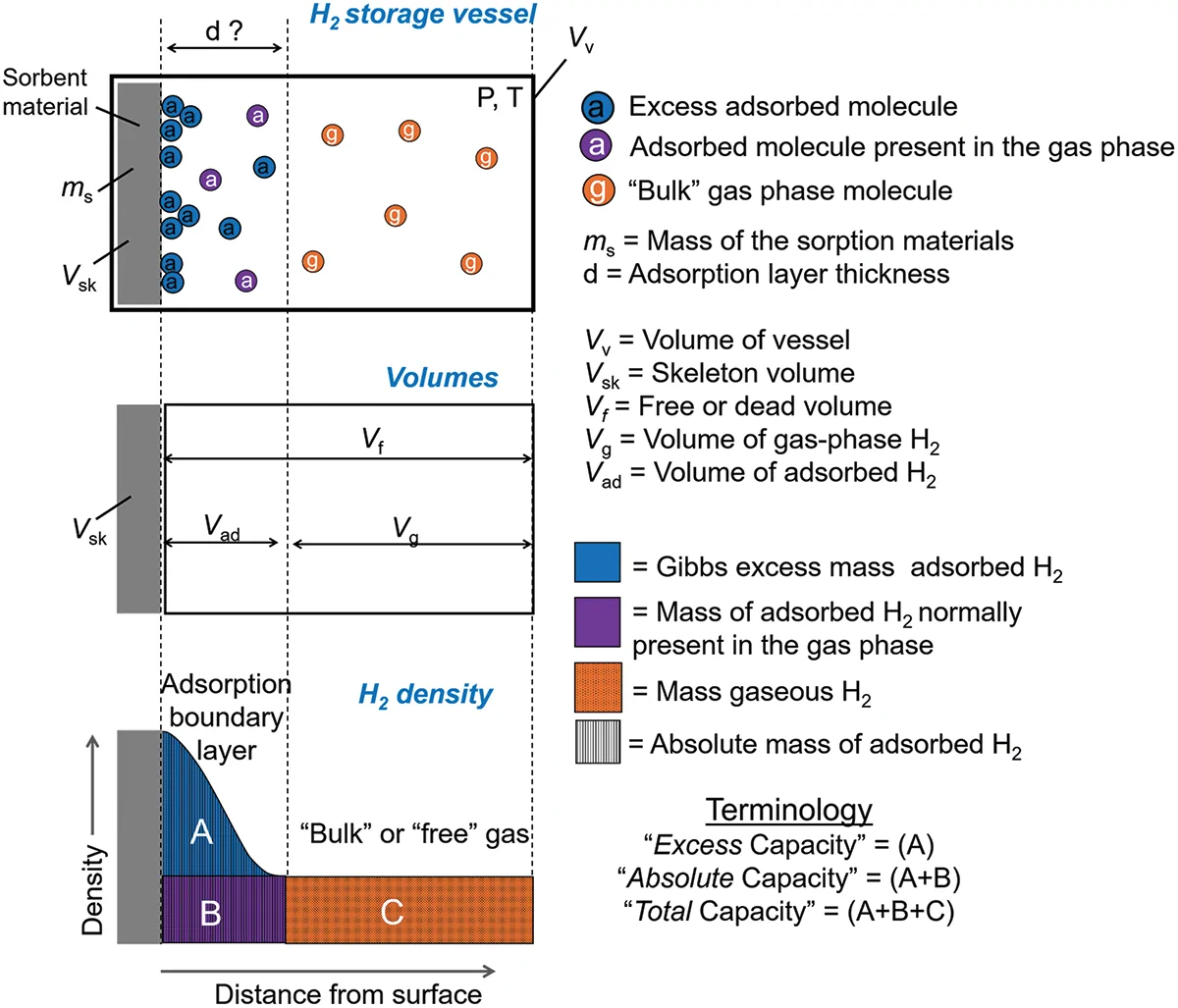
Illustration of the Gibbs surface excess mass for physisorption material in a storage vessel. Reproduced from Figure 82 of the ‘best practices’ document, K. Gross, Ref [9].

### 3.1. *‘*Excess*’ capacity*

‘*Excess*’, or more formally ‘*Gibbs surface excess capacity*’, *m*_ex_, simply represents the hydrogen molecules that are present as the adsorbate at the pore-surface, represented as region A in Figure 2. The expression for the ‘*exces*s’ amount is,

(10) $$m_{\mathrm{ex}}=\;m^{*}-\;\rho_{g}\left(V_{f}\right)=m^{*}-\;\rho_{g}\left(V_{v}-\;V_{\mathrm{sk}}\right)$$

Here, m* is the total mass of hydrogen gas in the experimental vessel or system. *Excess* adsorption is basically directly measured in a standard manometric (Sieverts-type) and gravimetric instruments.

To illustrate this, let’s consider a single gas expansion step in a volumetric measurement. An initial dose of H_2_ in a reference vessel has a known mass, m* as determined by the temperature, pressure of the gas and volume of the reference cell. It is then expanded into the sample vessel by opening a valve. The total free volume (or ‘dead volume’) is given by Equation (9), where *V*_sk_ is the skeletal volume of the sample volume determined initially from helium expansion measurements, and *V*_v_ is the total calibrated volume of the instrument. After the gas is dosed into the sample vessel, one waits for the system to reach equilibrium state, and then measures the temperature and pressure of the free gas. The bulk gas density $\;\rho_{g}\left(H_{2}\right)$ is determined using a real gas equation of state, and the *excess* adsorption amount is calculated using Equation (10).

The *maximum excess capacity* is a material property that specifies the maximum amount of H_2_ that can be adsorbed by a material. In general, excess adsorption can increase with decreasing temperature to 77 K or below, reach a maximum at high pressure, and then decrease as the bulk-gas density increases, thereby reducing the density excess relative to the bulk reference state as illustrated in Figure 3.

**Figure 3.**
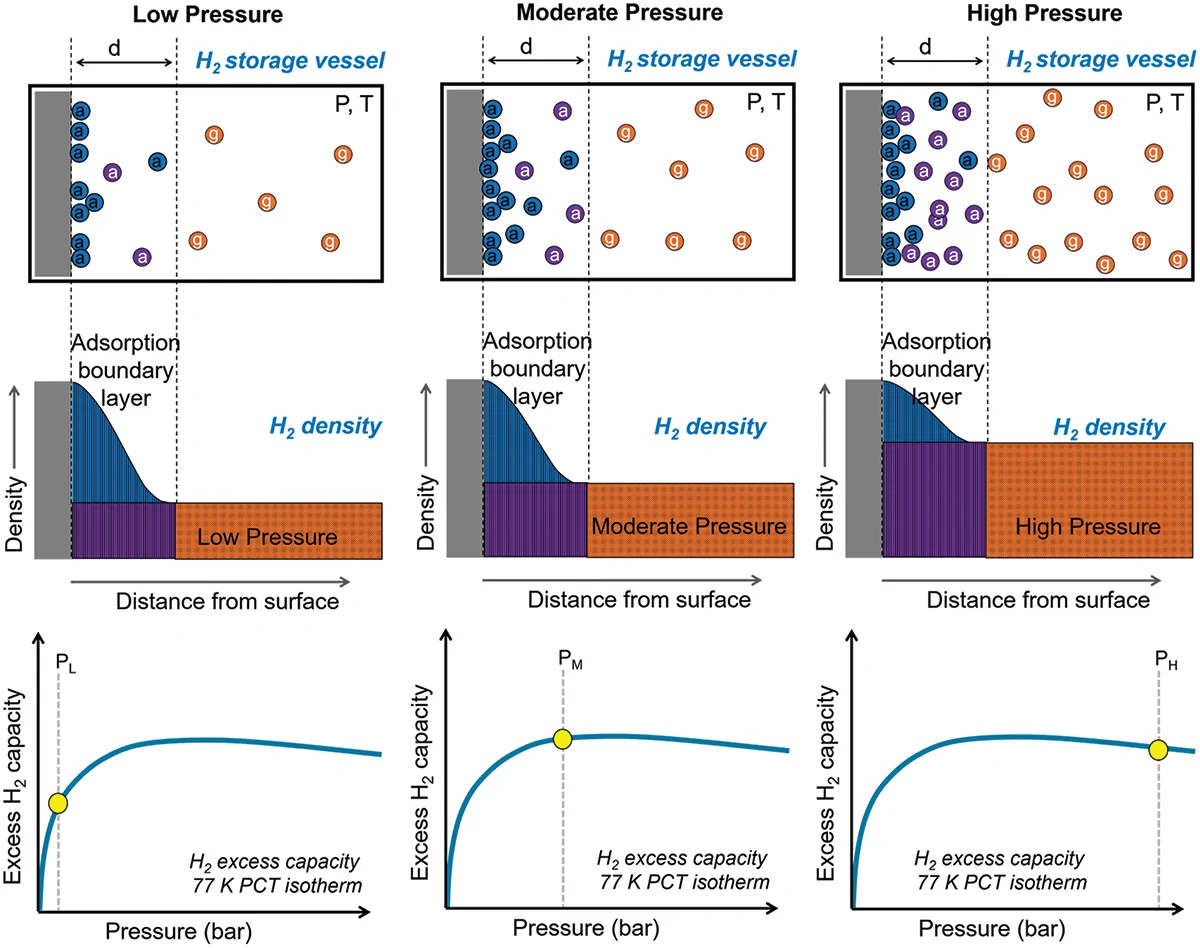
Illustration of the shape of the excess capacity curve for physisorption material at 77 K. Reproduced from Figure 86 of the ‘best practices’ document, K. Gross, Ref [9].

### 3.2. ‘Absolute*’ capacity*

‘*Absolute*’ capacity, $m_{\mathrm{abs}}$, is a conceptual construct based on the hypothetical concept of a two-dimensional surface layer of thickness ‘*d’*, in which adsorbed gas resides. By definition, $m_{\mathrm{abs}}$ counts all hydrogen molecules residing within the adsorption volume $V_{\mathrm{ad}}$ and represented by region A + B in Figure 2, and is expressed with the below formula:

(11) $$m_{\mathrm{abs}}=\;m_{\mathrm{ex}}+V_{\mathrm{ad}}\times \rho_{g}\left(P,\;T\right)$$

*Absolute* uptake is not directly measurable because its conversion from Gibbs *excess* requires an assumed adsorption-phase volume, *V*_ads_. The resulting absolute uptake is widely used in adsorption models, including Langmuir- and BET-type descriptions [7]. In narrow micropores (<2 nm), adsorption potentials from opposing walls overlap strongly, and *V*_ads_ is sometimes approximated by the intracrystalline pore volume, *V*_pore_ [14]. However, the location of an adsorption-phase boundary is not uniquely defined; consequently, absolute uptake is model dependent. Any approximation that equates *V*_ads_ with *V*_pore_ should therefore be stated explicitly.

### 3.3. *‘*Total*’ capacity*

*Total* capacity counts all hydrogen associated with the specified material control volume, including the Gibbs *excess* contribution and the compressed hydrogen occupying the gas-accessible volume. In Figure 2, this corresponds to regions A + B + C [7–9]. *Total* hydrogen is therefore the most directly relevant material-level quantity for evaluating the inventory of a vessel filled with an adsorbent, provided that the associated control volume is explicitly defined.

(12) $$m_{\mathrm{total}}=m_{\mathrm{ex}}+V_{\varphi }\times \rho_{g}\left(H_{2},P,T\right)$$

Here $V_{\varphi }$ is the specified gas-accessible control volume, and this must be stated clearly, otherwise, the definition of ‘total’ capacity is incomplete. The various materials’ capacity definitions for porous adsorbents are depicted schematically in Figure 4 for general understanding.

**Figure 4.**
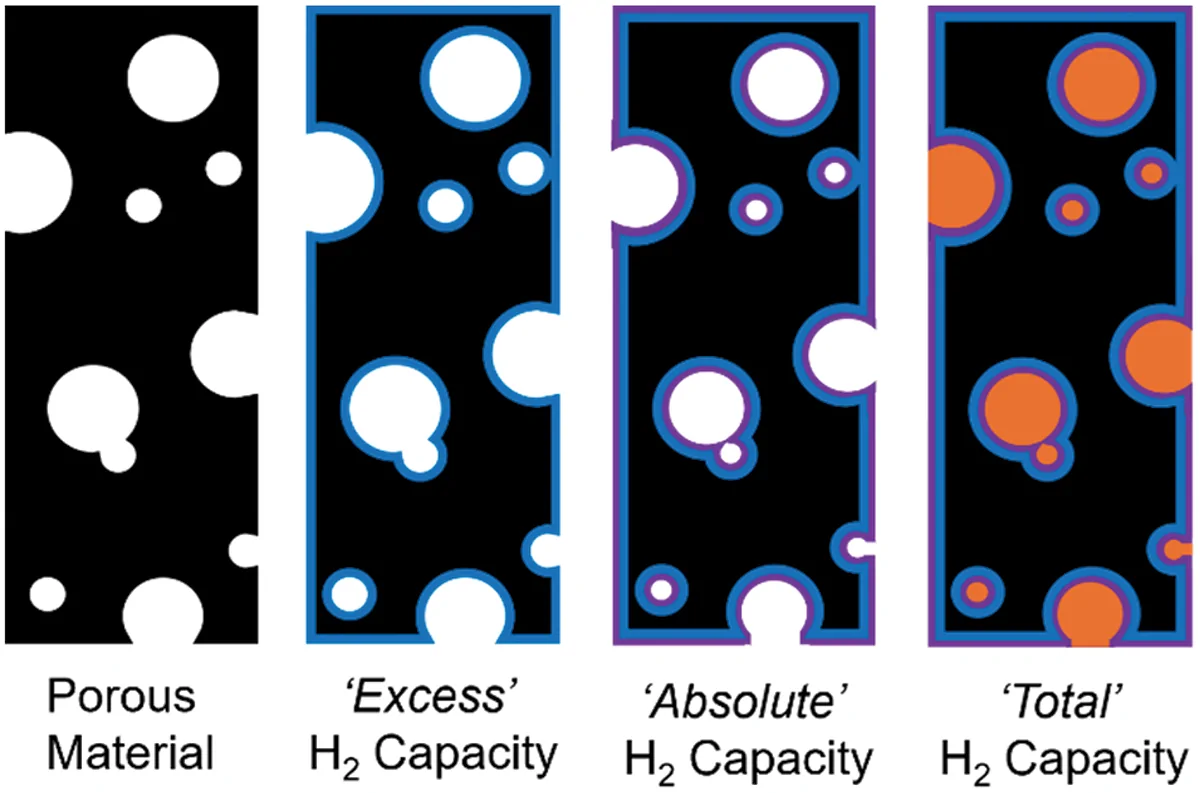
Illustration of the ‘*excess* H_2_ storage capacity’, ‘*absolute* H_2_ storage capacity’ and ‘*total* H_2_ storage capacity’ at a materials level. Reproduced from Figure 91 of the ‘best practices’ document, K. Gross, Ref [9]. The ‘blue’ covered region indicates ‘*excess*’, ‘blue + purple’ colored region indicates ‘*absolute*’, and ‘blue + purple + orange’ colored region indicates ‘*total*’ H_2_ storage capacity of the porous material.

#### 3.3.1. Total *gravimetric capacity and volumetric capacity*

##### 3.3.1.1. Total gravimetric storage capacity

To mention the hydrogen storage property of an adsorbent, two primary metrics are used: gravimetric and volumetric uptake. The experimentally determined Gibbs *excess* uptake is typically normalized by the mass of adsorbent sample (in g) and represented as *n*_ex_ (in mmol or in mg). The *total* gravimetric uptake is then calculated from the following equation and is equal to the *excess* uptake plus the amount of hydrogen that would occupy the entirety of the gas-accessible volume of the system, *V*_void_.

(13) $$n_{\mathrm{total}}=n_{\mathrm{ex}}+V_{\mathrm{void}}\times \rho_{g}$$

##### 3.3.1.2. Total volumetric storage capacity

The *total* volumetric uptake, *n*_total, vol._, is particularly important for applications in which the storage volume is constrained, such as onboard vehicles. *n*_total,vol._ can be determined by multiplying the total gravimetric uptake by the adsorbent density. Consequently, the density and control volume used in the conversion must be stated explicitly. For the packed-bed formulation adopted here, at least two of the three volumes (*V*_sk_, *V*_pk_, *V*_void_) are necessary to define or otherwise be obtained prior to the evaluation using Equations (6)–(8).

The first calculation method is referred to as ‘common’ approximation as per Samantaray *et al*., where experimentally measured *ρ*_pk_ of the sample and pore volume *V*_pore_ are combined to determine the total volumetric uptake as follows [8]:

(14) $$n_{\mathrm{total},\mathrm{vol}.1}=\left(n_{\mathrm{ex}}+\rho_{g}V_{\mathrm{pore}}\right)\times \rho_{\mathrm{pk}}$$

Although this approach is commonly used, it can create confusion between ‘total’ but perform ‘absolute’ capacity calculations, which used both assumption of *V*_ads_ or *V*_void_ close to *V*_pore_, and critically derived from N_2_ adsorption isotherms obtained at 77 K. Notably, the obtained *V*_pore_ varies substantially depending on the types of isotherm (Type 1, Type IV, etc.); the consideration of micropores or total pores (including micropores, mesopores, and/or interparticle pores, etc.); and the respective determination techniques (for instance, the Gurvich rule, Dubinin–Radushkevich (DR) method, Non-Local Density Functional Theory (NLDFT) and the *t*-plot method) [16–18]. Accordingly, the pore-volume definition and analysis method should always be stated, and an H_2_-relevant accessible volume should be used when available.

In the second calculation method, referred to as ‘skeletal’ approximation, the measured *ρ*_pk_ and *ρ*_sk_ of the sample are combined to get the gas accessible *V*_void_ using Equation (7) and the *total* uptake is reported as follows:

(15) $$n_{\mathrm{total},\mathrm{vol}.\;2}=\left(n_{\mathrm{ex}}+\rho_{g}\left(\frac{1}{\rho_{\mathrm{pk}}}-\frac{1}{\rho_{\mathrm{sk}}}\right)\right)\times \rho_{\mathrm{pk}}$$

In the third approach, the uptake referred to as ‘swollen’ approximation, *V*_pore_ and *ρ*_sk_ are combined as follows:

(16) $$n_{\mathrm{total},\mathrm{vol}.\;3}=\left(n_{\mathrm{ex}}+\rho_{g}V_{\mathrm{pore}}\right)\left(\frac{1}{V_{\mathrm{pore}}+{\rho_{\mathrm{sk}}}^{-1}}\right)$$

If the three determined volumes *V*_sk_, *V*_pk_, *V*_void_ are internally consistent, then the results from Equations (14)–(16) should yield the same results. In practice, these quantities are obtained from independent measurements that rely on different experimental assumptions, and substantial discrepancies can therefore arise, particularly for powders, as illustrated in Tables 1 and 2.

**Table 2.** Representative measurements and methods of calculation of volumetric H_2_ storage calculations on MOF-5.

Sample	Form	Calculation	$n_{\mathrm{total},\mathrm{vol}.\;}$ at 77 K, 100 bar (g L^−1^)	Reference
MOF-5	Powder^2,5^	$n_{\mathrm{total},\mathrm{vol}.\;1}$(Equation (14))	20.5	[8]
	Powder^2,4^	$n_{\mathrm{total},\mathrm{vol}.\;2}$ (Equation (15))	39.7	[8]
	Powder^4,5^	$n_{\mathrm{total},\mathrm{vol}.\;3}$ (Equation (16))	52.9	[8]
	Powder^6^	$n_{\mathrm{total},\mathrm{vol}.\;4}$ (Equation (17))	56.5	[8]
	Powder^1^	$n_{\mathrm{total},\mathrm{vol}.\;2}$ (Equation (15))	36.3	[8]
	Powder^2^	$n_{\mathrm{total},\mathrm{vol}.\;2}$ (Equation (15))	39.7	[8]
MOF-5/ENG	Pellet^3^	$n_{\mathrm{total},\mathrm{vol}.\;2}$ (Equation (15))	45.2	[8,14]
	Pellet^3^	$n_{\mathrm{total},\mathrm{vol}.\;2}$ (Equation (15))	41.7	[8,14]

^1^Bulk density measured by manual tapping; ^2^Packing density measured by jolting volumetry; ^3^Packing density measured by calipers or ruler; ^4^Skeletal density measured by He pycnometry at 298 K; ^5^Pore volume measured by N_2_ adsorption at 77 K; ^6^Packing density and $\chi_{\mathrm{void}}$ calculated from the CIF using Zeo++.

##### 3.3.1.3. *Crystal-limit* total *volumetric uptake*

Therefore, to estimate the theoretical limit of the *total* volumetric uptake, a gravimetric excess adsorption isotherm measured on a high-quality powder can be converted using ideal crystallographic properties. This treatment uses the crystal density, and the crystallographically calculated gas-accessible and void fraction, and therefore represents an upper bound before real packing losses are introduced. The crystal-limit *total* volumetric uptake can be expressed as:

(17) $$n_{\mathrm{total},\mathrm{vol}.,4}=\rho_{\mathrm{crys}}n_{\mathrm{ex}}+\rho_{g}\times \chi_{\mathrm{void},\mathrm{crys}}$$

#### 3.3.2. System-level total volumetric uptake

Although much of the literature focuses on material-level hydrogen adsorption (particle or packed adsorbents), DOE onboard-storage targets are defined at the system-level and include storage medium, the pressure of vessel, thermal management hardware and other balance-of-plant components [22,23]. A detailed discussion of complete-system engineering is beyond the scope of this Perspective. However, if the objective is to count the *total* hydrogen physically present within a calibrated vessel volume, including headspace outside the packed bed, the vessel-level inventory must include that additional gas volume explicitly:

(18) $$m_{\mathrm{total},\mathrm{vessel}}=m_{s}n_{\mathrm{ex}}+\rho_{g}\left(V_{v}-V_{\mathrm{sk}}\right)$$

## 4. Working capacity

As defined in practical hydrogen-storage targets, storage capacities are generally expressed in terms of ‘working’ or ‘usable’ or ‘deliverable’ capacity, referring to the amount of hydrogen that can be effectively delivered from a filled, high-pressure storage tank to the required low-pressure delivery condition, as illustrated in Figure 5 [8,19].

**Figure 5.**
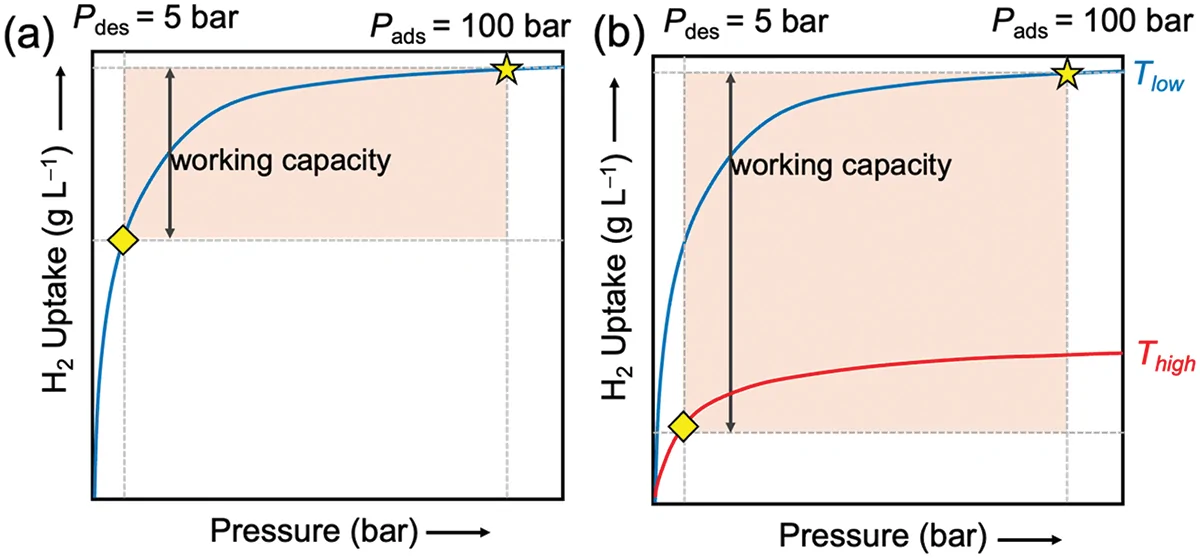
Illustration of working volumetric capacity for (a) pressure swing and (b) temperature-pressure swing storage systems. The total volumetric isotherms shown in blue and red curves correspond to high and low temperatures, respectively. The charged state is represented by a star, whereas the discharged state is represented by diamonds. Reproduced from Ref [11].

For porous adsorbents, a pressure window of 100 bar (charged state) to 5 bar (discharged state) for delivery is commonly used as a practical benchmark to evaluate deliverable hydrogen storage. Many rigid porous adsorbents typically exhibit classical Type-I or Langmuir-type adsorption isotherms, characterized by rapid gas uptake at low pressure followed by gradual saturation at higher pressure. This behavior limits the working capacity under a simple *P*-swing technique, since much of the stored hydrogen remains adsorbed and cannot be released within the defined pressure window (Figure 5(a)) [19]. One effective strategy to overcome this limitation is to combine pressure swing with temperature swing (*PT*-swing) operation (Figure 5(b)) [8,20]. In this *PT*-swing approach, the initial adsorption occurs at a lower temperature (*T*_low_), and the desorption is subsequently driven by warming the system to a high temperature (*T*_high_), while maintaining the same working pressure of 100 to 5 bar. This dual swing can increase the working capacity by reducing the amount of hydrogen retained by the adsorbent at the discharge condition.

However, the reported deliverable capacity remains highly dependent on the selected pressure and temperature window. Many studies emphasize *P*-swing at 77 K or *PT*-swing from 100 bar/77 K to 5 bar/298 K, but these conditions do not necessarily identify the optimum operating window for every adsorbent. Therefore, hydrogen uptake and estimation of deliverable capacities at intermediate temperatures, such as 120 K or 160 K, should be checked and compared against compressed hydrogen under the same operating condition [8,21].

## 5. ‘Unit’ of gravimetric uptake

While reporting, the common practice is to represent *n*_ex_ in weight percentage (wt%) and is expressed as: $\mathrm{wt}\%=\;m_{H_{2}}/\left(m_{\mathrm{host}}+m_{H_{2}}\right)\times 100\%$, where $m_{H_{2}}$ is the mass of adsorbed H_2_ and $m_{\mathrm{host}}$ is the mass of adsorbent. However, many researchers omit the $m_{H_{2}}$ from the denominator, which creates a discrepancy while comparing the data. This difference becomes especially significant for materials with high H_2_ uptake capacities, particularly at 77 K. Therefore, we caution all researchers to be clear about the definition of wt% and indicate whether the denominator is taken to be the initial mass of the adsorbent or that of the adsorbent plus adsorbed H_2_ employed. For this reason, the use of wt% has been discouraged in favor of mmol g^−1^, which always refers to grams of sorbent material alone [2,8].

### 5.1. Key considerations for hydrogen adsorption measurements

As discussed above, hydrogen adsorption equilibria in porous adsorbents are commonly measured under isothermal conditions using either a gravimetric (with a balance) or volumetric (with a Sieverts apparatus) technique. During measurement, data analysis, and reporting, several points require careful attention.

- When researchers report the final normalized *n*_ex_ as a weight percentage (wt%), we caution all researchers to be clear about the definition of wt% employed and recommend against its use in favor of mmol g^−1^ (which always refers to grams of sorbent material alone).
- Regardless of the method employed, *ρ*_sk_ must be determined and reported. This is typically performed using helium pycnometry at elevated pressures and room temperature. However, helium adsorption at room temperature may be non-negligible, causing the measured *ρ*_sk_ to be higher than the actual value. To mitigate this effect, it is recommended to measure *ρ*_sk_ at elevated temperatures, where helium adsorption is negligible.
- Caution is needed when using the terms ‘absolute’ or ‘total’ uptake.
- Density terms must be clearly distinguished because volumetric uptake is directly related to the density of adsorbents. Authors should report the skeletal density (*ρ*_sk_), crystallographic or single crystal density (*ρ*_crys_), packed density for powders (*ρ*_pk_), and packed or envelope density for pellets or monoliths (*ρ*_env_).
- While measuring the working capacity of porous solids, it is recommended to measure *PT*-swing rather than relying solely on P-swing measurements at several temperatures such as 77, 120, 160, and 298 K to find the best-performing condition with respect to compressed gas storage under the same operating conditions.

Overall, we encourage researchers to include a reporting checklist as listed in Table 3, when presenting the hydrogen storage performance of porous adsorbents. A flow-chart to assess the volumetric hydrogen uptake by a volumetric PCT instrument is mentioned herein for readers’ clarity and better understanding in Figure 6.

**Figure 6.**
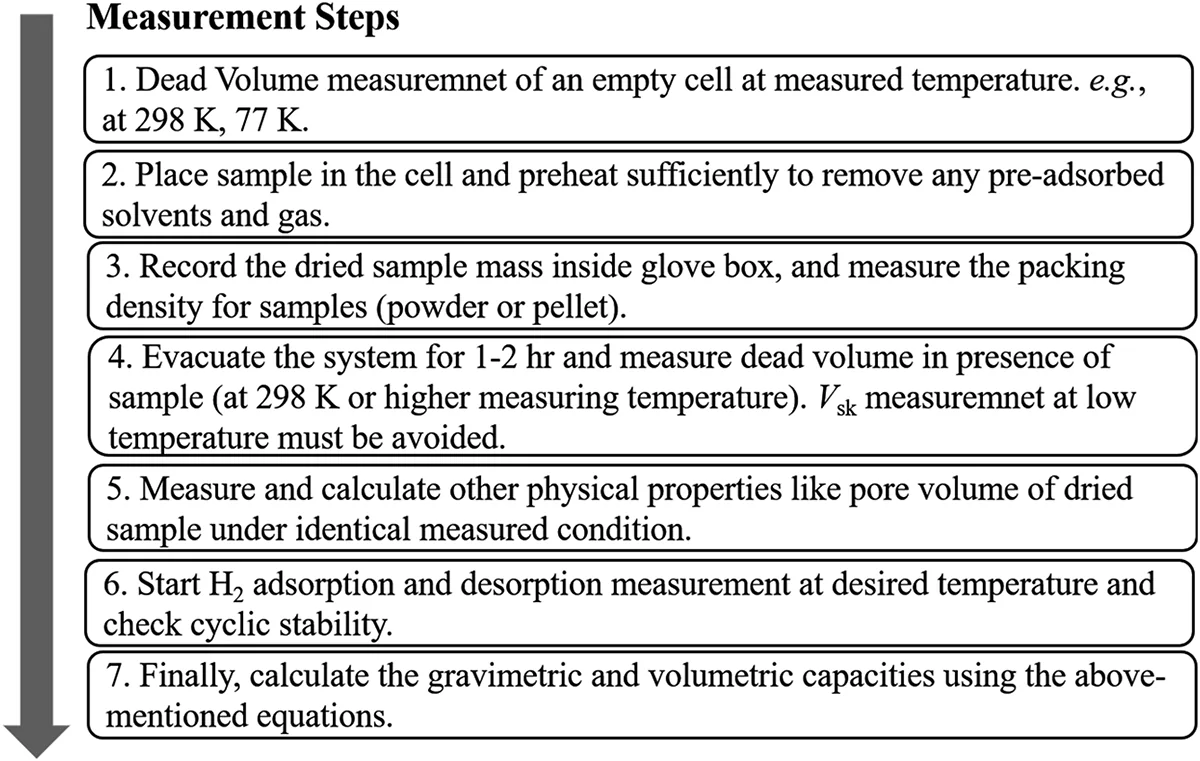
Schematic of flow-chart of a general hydrogen adsorption isotherm measurement using volumetric PCT apparatus.

**Table 3.** Recommended reporting checklist for hydrogen storage measurements in porous adsorbents.

Sample form	Sample mass (g)	Skeleton density (g cm^−3^)	Single crystal density (g cm^−3^)	Packing density (g cm^−3^)	Pore Volume (cm^3^ g^−1^)	Void Volume (cm^3^ g^−1^)	Gravimetric Excess (mg g^−1^/ mmol g^−1^/ wt%)	Absolute uptake/Total uptake
Powder/ Pellet/Monolith	*m*_s_^1^	*ρ*_sk_^2^	*ρ*_crys_^3^	*ρ*_pk_^4^	*V*_pore_^5^	*V*_void_^6^	*n*_ex_^7^	*n*_total_^8^

^1^*m*_s_: Final mass of the sample after degassing the adsorbent.

^2^*ρ*_sk_: Skeletal density measured by helium pycnometry or using a volumetric PCT instrument at elevated pressures and temperatures.

^3^*ρ*_crys_: Crystallographic or single‑crystal density obtained from structural data.

^4^*ρ*_pk_: Packing density measured by jolting volumetry or tapping method, calipers, or ruler.

^5^*V*_pore_: Micropores or total pore volume obtained from N_2_ adsorption isotherm analysis, with the method clearly stated.

^6^*V*_void_: Gas-accessible void volume of powder bed, pellet, or monolith, calculated from relative density equations.

^7^*n*_ex_: Gravimetric excess uptake, preferably reported in mmol g^−1^ or mg g^−1^.

^8^*n*_abs_/n_total_: Absolute or total H_2_ uptake, based on density (*ρ*_crys_, *ρ*_bulk_, *ρ*_env_) and accessible pores (*V*_ads_ or *V*_void_).

In summary, adsorption of hydrogen in a porous solid remains an active area of research; however, hydrogen counting in such a system is not straightforward. Because excess, absolute, total, and volumetric capacities depend on different assumptions, clear reporting is essential. Although DOE targets are defined at the full-system level, including tank and balance-of-plant components, this perspective focuses on the material matrix to clarify how hydrogen uptake should be counted and compared in porous adsorbents. At the same time, application-oriented validation using direct measurements of stored and deliverable hydrogen, for example, through water-displacement or equivalent gas-release measurements, remains important for confirming the practical relevance of reported capacities and connecting material-level metrics to real storage performance.

## Abbreviations

DOE: Department of Energy
FOM: Figure of merit
EOS: Equation of state
PCT: Pressure-composition-temperature
*V*_sk_: Skeletal volume
*V*_crys_: Crystal volume
*V*_env_: Particle envelope volume
*V*_pk_: Packing or bed volume
*V*_v_: Calibrated internal vessel volume
*V*_pore_: Gas-accessible intraparticle pore volume
*V*_inter-particle_: Interparticle void volume
*V*_void_: Gas accessible void volume
*χ*_void_: Gas accessible void fraction
*V*_ads_: Assumed adsorption-phase volume
*V*_f_: Free or dead volume of instrument
*ρ*_sk_: Skeletal density
*ρ*_crys_: Ideal crystal density
*ρ*_env_: Envelope density
*ρ*_pk_: Packing density
*ρ*_*g*_(P, T): H_2_ mass density from a real-gas EOS
*m*_ex_: Mass of *excess* H_2_ adsorbed
*n*_ex_: Gibbs *excess* uptake, gravimetric
*n*_abs_: *Absolute* uptake, gravimetric
*n*_total_: *Total* uptake, gravimetric
*n*_total, vol_: *Total* volumetric uptake

## References

[1] U.S. Department of Energy. DOE technical targets for onboard hydrogen storage for light-duty vehicles [Internet]. Washington (DC): U.S. Department of Energy; [cited 2026 Aug 10]. Available from: https://www.energy.gov/cmei/fuels/doe-technical-targets-onboard-hydrogen-storage-light-duty-vehicles

[2] Suh MP, Park HJ, Prasad TK, et al. Hydrogen storage in metal-organic frameworks. Chem Rev. 2012;112(2):782–11. doi: 10.1021/cr200274s 22191516

[3] Balderas-Xicohténcatl R, Schlichtenmayer M, Hirscher M. Volumetric hydrogen storage capacity in metal-organic frameworks. Energy Technol. 2018;6(3):578–582. doi: 10.1002/ente.201700636

[4] Villajos JA. Experimental volumetric hydrogen uptake determination at 77 K of commercially available metal-organic framework materials. C. 2022;8(1):5. doi: 10.3390/c8010005

[5] Chen J, Tang Z, Zhu D, et al. Three-dimensional covalent organic framework for efficient hydrogen storage through polarization-wall engineering. Nano Lett. 2025;25(15):6268–6275. doi: 10.1021/acs.nanolett.5c00934 40178885

[6] Han SS, Furukawa H, Yaghi OM, et al. Covalent organic frameworks as exceptional hydrogen storage materials. J Am Chem Soc. 2008;130(35):11580–11581. doi: 10.1021/ja803247y 18683924

[7] Parilla PA, Hurst KE, Gennett T, et al. Recommended volumetric capacity definitions and protocols for accurate, standardized and unambiguous metrics for hydrogen storage materials. Appl Phys A. 2016;122(3):201. doi: 10.1007/s00339-016-9654-1

[8] Samantaray SS, Putnam ST, Stadie NP. Volumetrics of hydrogen storage by physical adsorption. Inorganics. 2021;9(6):45. doi: 10.3390/inorganics9060045

[9] Gross KJ, Carrington KR, Barcelo S, et al. Recommended best practices for the characterization of storage properties of hydrogen storage materials [Internet]. Golden (CO): National Renewable Energy Laboratory; 2016 [cited 2026 Aug 10]. Available from: https://www.energy.gov/documents/bestpracticeshydrogenstoragepdf

[10] Furukawa H, Miller MA, Yaghi OM. Independent verification of the saturation hydrogen uptake in MOF-177 and establishment of a benchmark for hydrogen adsorption in metal-organic frameworks. J Mater Chem. 2007;17(30):3197–3204. doi: 10.1039/b703608f

[11] Madden DG, O’Nolan D, Rampal N, et al. Densified HKUST-1 monoliths as a route to high volumetric and gravimetric hydrogen storage capacity. J Am Chem Soc. 2022;144(30):13729–13739. doi: 10.1021/jacs.2c04608 PMC9354247 35876689

[12] Purewal J, Veenstra M, Tamburello D, et al. Estimation of system-level hydrogen storage for metal-organic frameworks with high volumetric storage density. Int J Hydrogen Energy. 2019;44(29):15135–15145. doi: 10.1016/j.ijhydene.2019.04.082

[13] Ming Y, Purewal J, Liu D, et al. Thermophysical properties of MOF-5 powders. Microporous Mesoporous Mater. 2014;185:235–244. doi: 10.1016/j.micromeso.2013.11.015

[14] Purewal J, Liu D, Sudik A, et al. Improved hydrogen storage and thermal conductivity in high-density MOF-5 composites. J Phys Chem C. 2012;116(38):20199–20212. doi: 10.1021/jp305524f

[15] Purewal JJ, Liu D, Yang J, et al. Increased volumetric hydrogen uptake of MOF-5 by powder densification. Int J Hydrogen Energy. 2012;37(3):2723–2727. doi: 10.1016/j.ijhydene.2011.03.002

[16] Ongari D, Boyd PG, Barthel S, et al. Accurate characterization of the pore volume in microporous crystalline materials. Langmuir. 2017;33(51):14529–14538. doi: 10.1021/acs.langmuir.7b01682 PMC5745516 28636815

[17] Ravikovitch PI, Neimark AV. Characterization of nanoporous materials from adsorption and desorption isotherms. Colloids Surf Physicochem Eng Asp. 2001;187–188:11–21. doi: 10.1016/S0927-7757(01)00614-8

[18] Seaton NA, Walton JPRB, Quirke N. A new analysis method for the determination of the pore size distribution of porous carbons from nitrogen adsorption measurements. Carbon. 1989;27(6):853–861. doi: 10.1016/0008-6223(89)90035-3

[19] García-Holley P, Schweitzer B, Islamoglu T, et al. Benchmark study of hydrogen storage in metal-organic frameworks under temperature and pressure swing conditions. ACS Energy Lett. 2018;3(3):748–754. doi: 10.1021/acsenergylett.8b00154

[20] Sutton AL, Mardel JI, Hill MR. Metal-organic frameworks (MOFs) as hydrogen storage materials at near-ambient temperature. Chem Eur J. 2024;30(44):e202400717. doi: 10.1002/chem.202400717 38825571

[21] Evans HA, Yildirim T, Peng P, et al. Hydrogen storage with aluminum formate, ALF: experimental, computational, and technoeconomic studies. J Am Chem Soc. 2023;145(40):22150–22157. doi: 10.1021/jacs.3c08037 37767573

[22] Duncan J, Sengupta D, Bose S, et al. Defect-induced confinement in zirconium metal-organic frameworks for enhanced hydrogen adsorption. Sustain Chem Environ. 2023;3:100032. doi: 10.1016/j.scenv.2023.100032

[23] Connolly BM, Aragones-Anglada M, Gandara-Loe J, et al. Tuning porosity in macroscopic monolithic metal-organic frameworks for exceptional natural gas storage. Nat Commun. 2019;10(1):2345. doi: 10.1038/s41467-019-10185-1 PMC6538620 31138802

